# Genome-Wide Scan Identifies *TNIP1*, *PSORS1C1*, and *RHOB* as Novel Risk Loci for Systemic Sclerosis

**DOI:** 10.1371/journal.pgen.1002091

**Published:** 2011-07-07

**Authors:** Yannick Allanore, Mohamad Saad, Philippe Dieudé, Jérôme Avouac, Jorg H. W. Distler, Philippe Amouyel, Marco Matucci-Cerinic, Gabriella Riemekasten, Paolo Airo, Inga Melchers, Eric Hachulla, Daniele Cusi, H.-Erich Wichmann, Julien Wipff, Jean-Charles Lambert, Nicolas Hunzelmann, Kiet Tiev, Paola Caramaschi, Elisabeth Diot, Otylia Kowal-Bielecka, Gabriele Valentini, Luc Mouthon, László Czirják, Nemanja Damjanov, Erika Salvi, Costanza Conti, Martina Müller, Ulf Müller-Ladner, Valeria Riccieri, Barbara Ruiz, Jean-Luc Cracowski, Luc Letenneur, Anne Marie Dupuy, Oliver Meyer, André Kahan, Arnold Munnich, Catherine Boileau, Maria Martinez

**Affiliations:** 1 Université Paris Descartes, Rhumatologie A, INSERM, U1016, Hôpital Cochin, APHP, Paris, France; 2 INSERM, U781, Université Paris Descartes, Hôpital Necker, Paris, France; 3 INSERM, U563, CHU Purpan, Université Paul Sabatier, Toulouse, France; 4 Université Paris Diderot, Rhumatologie, INSERM, U699, Hôpital Bichat Claude-Bernard, Paris, France; 5 Department for Internal Medicine 3 and Institute for Clinical Immunology Friedrich-Alexander-University Erlangen-Nuremberg, Nuremberg, Germany; 6 INSERM, U744, Institut Pasteur de Lille, Université de Lille Nord, Lille, France; 7 Department of Biomedicine, Division of Rheumatology AOUC, Denothe Centre, University of Florence, Florence, Italy; 8 Department of Rheumatology and Clinical Immunology, Charité University Hospital, Berlin, Germany; 9 Rheumatology and Clinical Immunology, Spedali Civili, Brescia, Italy; 10 Clinical Research Unit for Rheumatology, University Medical Center, Freiburg, Germany; 11 Lille II University, Internal Medecine Department, Lille, France; 12 University of Milano, Department of Medicine, Surgery, and Dentistry San Paolo School of Medicine; 13 Genomics and Bioinformatics Platform, Fondazione Filarete, Milan, Italy; 14 Institute of Epidemiology I, Helmholtz Zentrum München – German Research Center for Environmental Health, Neuherberg, Germany; 15 Institute of Medical Informatics, Biometry, and Epidemiology, Chair of Epidemiology, Ludwig-Maximilians-Universität and Klinikum Grosshadern, Munich, Germany; 16 Department of Dermatology, University of Cologne, Köln, Germany; 17 Université Pierre et Marie Curie, Service de Médecine Interne, Hôpital Saint Antoine, Paris, France; 18 Rheumatology Unit, University of Verona, Verona, Italy; 19 INSERM, U618, IFR 135, CHU Bretonneau, Tours, France; 20 Department of Rheumatology and Internal Medicine, Medical University of Bialystok, Bialystok, Poland; 21 Department of Clinical and Experimental Medicine, Rheumatology Unit, Second University of Naples, Naples Italy; 22 Université Paris Descartes, Médecine Interne, Hôpital Cochin, APHP, Paris, France; 23 Department of Immunology and Rheumatology, University of Pécs, Pécs, Hungary; 24 Institute of Rheumatology, School of Medicine, University of Belgrade, Belgrade, Serbia; 25 Kos Genetic SRL, Milano, Italy; 26 Institute of Genetic Epidemiology, Helmholtz Zentrum München – German Research Center for Environmental Health, Neuherberg, Germany; 27 Institute of Medical Informatics, Biometry, and Epidemiology, Chair of Epidemiology and Chair of Genetic Epidemiology, Ludwig-Maximilians-Universität and Department of Medicine I, University Hospital Grosshadern, Ludwig-Maximilians-Universität, Munich, Germany; 28 University of Giessen, Department of Rheumatology and Clinical Immunology Kerckhoff-Klinik, Bad Nauheim, Germany; 29 Division of Rheumatology, Department of Internal Medicine and Medical Specialities, University “Sapienza,” Rome, Italy; 30 INSERM, CIC3, CHU Grenoble, France; 31 INSERM, U897, Bordeaux, France; 32 Université Bordeaux Segalen, Bordeaux, France; 33 INSERM, U888, Hôpital de la Colombière, Montpellier, France; 34 Université Versailles-SQY, Laboratoire de Biochimie, d'Hormonologie et de Génétique Moléculaire, Hôpital Ambroise Paré, AP-HP, Boulogne, France; University of Oxford, United Kingdom

## Abstract

Systemic sclerosis (SSc) is an orphan, complex, inflammatory disease affecting the immune system and connective tissue. SSc stands out as a severely incapacitating and life-threatening inflammatory rheumatic disease, with a largely unknown pathogenesis. We have designed a two-stage genome-wide association study of SSc using case-control samples from France, Italy, Germany, and Northern Europe. The initial genome-wide scan was conducted in a French post quality-control sample of 564 cases and 1,776 controls, using almost 500 K SNPs. Two SNPs from the MHC region, together with the 6 loci outside MHC having at least one SNP with a P<10^−5^ were selected for follow-up analysis. These markers were genotyped in a post-QC replication sample of 1,682 SSc cases and 3,926 controls. The three top SNPs are in strong linkage disequilibrium and located on 6p21, in the *HLA-DQB1* gene: rs9275224, P = 9.18×10^−8^, OR = 0.69, 95% CI [0.60–0.79]; rs6457617, P = 1.14×10^−7^ and rs9275245, P = 1.39×10^−7^. Within the MHC region, the next most associated SNP (rs3130573, P = 1.86×10^−5^, OR = 1.36 [1.18–1.56]) is located in the *PSORS1C1* gene. Outside the MHC region, our GWAS analysis revealed 7 top SNPs (P<10^−5^) that spanned 6 independent genomic regions. Follow-up of the 17 top SNPs in an independent sample of 1,682 SSc and 3,926 controls showed associations at *PSORS1C1* (overall P = 5.70×10^−10^, OR:1.25), *TNIP1* (P = 4.68×10^−9^, OR:1.31), and *RHOB* loci (P = 3.17×10^−6^, OR:1.21). Because of its biological relevance, and previous reports of genetic association at this locus with connective tissue disorders, we investigated TNIP1 expression. A markedly reduced expression of the *TNIP1* gene and also its protein product were observed both in lesional skin tissue and in cultured dermal fibroblasts from SSc patients. Furthermore, TNIP1 showed *in vitro* inhibitory effects on inflammatory cytokine-induced collagen production. The genetic signal of association with *TNIP1* variants, together with tissular and cellular investigations, suggests that this pathway has a critical role in regulating autoimmunity and SSc pathogenesis.

## Author Summary

Systemic sclerosis (SSc) is a connective tissue disease characterized by generalized microangiopathy, severe immunologic alterations, and massive deposits of matrix components in the connective tissue. Epidemiological investigations indicate that SSc follows a pattern of multifactorial inheritance; however, only a few loci have been replicated in multiple studies. We undertook a two-stage genome-wide association study of SSc involving over 8,800 individuals of European ancestry. Combined analyses showed independent association at the known *HLA-DQB1* region and revealed associations at *PSORS1C1*, *TNIP1*, and *RHOB* loci, in agreement with a strong immune genetic component. Because of its biological relevance, and previous reports of genetic association at this locus with other connective tissue disorders, we investigated TNIP1 expression. We observed a markedly reduced expression of the gene and its protein product in SSc, as well as its potential implication in control of extra-cellular matrix synthesis, providing a new clue for a link between inflammation/immunity and fibrosis.

## Introduction

Systemic sclerosis (MIM181750) is a connective tissue disease characterized by generalized microangiopathy, severe immunologic alterations and massive deposits of matrix components in the connective tissue. Being an orphan disease, SSc presents a major medical challenge and is recognized as the most severe connective tissue disorder with high risk of premature deaths [1]. Epidemiological data on SSc vary in different parts of the world and depend on selection criteria for the study population. Inasmuch, the prevalence of the disease fluctuates across global regions and population-based studies result in higher prevalence than do hospital records-based studies. In North America, the prevalence of SSc has been reported as 0.7–2.8 per 10,000 in a Canadian study, whereas in the U.S. figures of 2.6 per 10,000 versus 7.5 per 10,000 were reported by medical records - versus population-based studies, respectively. In Europe, a prevalence of 1.6 per 10,000 was reported in Denmark, 3.5 per 10,000 in Estonia, 1.58 per 10,000 adults (95% confidence interval, 129–187) in Seine-Saint-Denis in France [2]–[4]. The risk of SSc is increased among first-degree relatives of patients, compared to the general population. In a study of 703 families in the US, including 11 multiplex SSc families, the familial relative risk in first-degree relatives was about 13, with a 1.6% recurrence rate, compared to 0.026% in the general population [5]. The sibling risk ratio was about 15 (ranging from 10 to 27 across cohorts). The only twin study reported to date included 42 twin pairs [6]. The data showed a similar concordance rate in monozygotic twins (4.2%, n = 24) and dizygotic twins (5.6%) (NS) and an overall cross-sectional concordance rate of 4.7%. However, concordance for the presence of antinuclear antibodies was significantly higher in the monozygotic twins (90%) than in the dizygotic twins (40%) suggesting that genetics may be important for the auto-immune part of the disease.

The aetiology of SSc is still unclear but some key steps have been described, in particular early endothelial damage and dysregulation of the immune system with abnormal autoantibody production [7]. At the cellular level, early events include endothelial injury and perivascular inflammation with the release of a large array of inflammatory mediators [8], [9]. In the advanced stage, a progressive activation of fibroblasts in the skin and in internal organs leads to hyperproduction of collagen and irreversible tissue fibrosis [9]. Epidemiological investigations indicate that SSc follows a pattern of multifactorial inheritance [10]. Previous candidate-gene association studies have only identified a handful of SSc risk loci, most contributing to the genetic susceptibility of other autoimmune diseases [9]–[16]. So far, two genome-wide association studies of SSc have been conducted [17], [18]. The studies differ according to the ancestry of the studied population (Korean vs US/European) and the genome-wide association data: map density (∼440 K vs 280 K SNPs) and sample size (∼700 vs ∼7300 subjects). They provided evidence of association with known MHC loci, but only one ‘new’ locus was identified at *CD247* in the US/European dataset, variants at *CD247* being known to contribute to the susceptibility of systemic lupus erythematosus [18].

The diagnosis of SSc is based on recognized clinical criteria established decades ago however, these do not include specific autoantibodies or recent tools for assessment of the disease [19], [20]. Therefore, phenotypic heterogeneity is a concern for SSc and genetic heterogeneity is also highly probable with regards to data obtained in other connective tissue disorders. Given these considerations, and previous findings in other autoimmune diseases, it is apparent that additional risk variants for SSc remain to be discovered. Therefore, to identify further common variants that contribute to SSc risk in the European population, we conducted a two-stage GWAS, in two case-control samples (total >8,800 subjects).

## Results/Discussion

**Figure 1:**
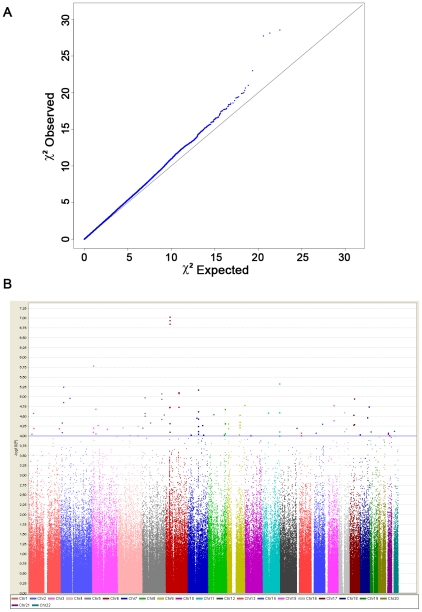
Genome-wide association results from the discovery phase. (A) Quantile-quantile plot for test statistics (logistic regression test) for 489,814 SNPs passing quality control. The plot shows a close match to the test statistics expected under the null distribution (λ = 1.03). (B) Manhattan plot representing the P values across the genome. The −log10 P of the logistic regression test (y axis) from 489,814 SNPs in 564 systemic sclerosis patients and 1,776 controls is plotted against its physical position (x-axis) on successive chromosomes. 90 SNPs with P<10^−4^ lie above the blue horizontal line and are listed in Table S1. Highly significant association was observed with SNPs within the MHC locus, including 1 SNPs that reached the conservative threshold for genome-wide significance (P<10^−7^).

We established a collaborative consortium including groups from 4 European countries (France, Italy, Germany and Eastern-Europe) from which we were able to draw upon a combined sample of over 8,800 subjects (before quality control) and conducted a two-stage genome-wide association study. In stage 1, we genotyped 1,185 samples on Illumina Human610-Quad BeadChip and genotypes obtained using the same chip from 2,003 control subjects were made available to us from the 3C study [21], [22]. After stringent quality control, we finally tested for association in stage-1, 489,814 autosomal SNPs in 2,340 subjects (564 cases and 1,776 controls) (Table 1). We tested for association between each SNP and SSc using the logistic regression association test, assuming additive genetic effects. The quantile-quantile plot and estimation of the genomic inflation factor (λ = 1.035) indicated minimal overall inflation (Figure 1A). The genome-wide logistic association results are presented in Figure 1B. Table S1 provides details for all SNPs with P<10^−4^, including one SNP exceeding P<10^−7^, the Bonferroni threshold for genome-wide significance. The three top SNPs were located on 6p21, in the *HLA-DQB1* gene: rs9275224, P = 9.18×10^−8^, OR = 0.69, 95%CI[0.60–0.79]; rs6457617, P = 1.14×10^−7^ and rs9275245, P = 1.39×10^−7^ (Figure 1B and Table 2). Several associated SNPs in *HLA-DQB1* have already been reported but rs6457617 was also identified as the most associated SNP in the previous US/European GWAS study [18]. Of note, the three SNPs in *HLA-DQB1* are in strong LD (r^2^>0.97). Within the MHC region, the next most associated SNP (rs3130573, P = 1.86×10^−5^, OR = 1.36[1.18–1.56]) is located in the psoriasis susceptibility 1 candidate 1 (*PSORS1C1*) gene (Table 2), a candidate gene for psoriasis [23]. Conditional analyses of susceptibility variants within MHC showed that there were two independent association signals at rs6457617 (*HLA-DQB1*) and at rs3130573 (*PSORS1C1*). Indeed, the association at PSORS1C1 remained significant (P<2.1×10^−5^) after controlling for the association at HLA-DQB1 and the association at HLA-DQB1 remained also significant (P<1.5×10^−7^) after controlling for the association at PSORS1C1 (Table S2).

**Table 1:** Description of the study population (post quality control samples).

	CASES					CONTROLS			TOTAL
	N	Mean age ±SD (years)	Female (%)	DcSSc	Topo+/ACA+ (%)	N	Mean age ±SD (years)	Female (%)	
**Stage 1: Discovery sample**									
	564	56.6±17.4	84%	34.8%	26% / 38.3%	488 (Genesys)	49.2±11.7	80%	
						1288 (3C)	73.99±5.6	65.5%	
Total DIS						1776	69±11.9	69.9%	2340
**Stage 2: Follow up samples**									
French	370	58.8±14.5	82.9%	28.7%	27.8% / 50.5%	1906	38.6±21.2	55.0%	
Italian	596	56.3±13.4	88.6%	25.4%	32.7% / 46%	490 (Italian network)	47.9±13.2	84.6%	
						721 (Hypergenes)	59.1±6.7	43.3%	
Eastern	151	53.1±12.4	93.6%	45.7%	24.6% / 13.8%	148	30.5±11.6	50%	
German	565	56.6±13.9	88.5%	35%	32.4% / 38.4%	180 (German network)	55±16.3	50%	
						481 (KORA study)	63.1±7.25	48.8%	
Total REP	1682	56.81±20.9	86.5%	30.5%	31.2% / 42.8%	3926	50.6±23.2	52.5%	5608

DcSSc: diffuse cutaneous systemic sclerosis; TOPO: anti topoisomerase I antibodies; ACA: anti-centromere antibodies.

**Table 2:** Genome-wide association and replication for systemic sclerosis risk variants.

				Discovery (564 cases/ 1,776 controls)	Replication (1,682 cases/ 3,926 controls)	Combined (2,246 cases/ 4,684 controls)
Chr. (closest gene)	Pos. (bp)	SNP	Minor/ Major	MAF Cases/ Controls	P	OR	95%CI	MAF Cases/ Controls	$P	OR	95%CI	**P	$P	OR	95%CI
**MHC loci**															
6p21(PSORS1C1)	31 214 247	rs3130573	G/A	0.391/ 0.321	1.86E-05	1.36	(1.18–1.56)	0.416/0.373	4.98E-03	1.13	(1.04–1.23)	4.8E-01	5.70E-10	1.25	(1.17–1.35)
6p21 (HLA-DQB1)	32 767 856	rs9275224	A/G	0.405/ 0.496	9.18E-08	0.69	(0.6–0.79)	&NA	-	-	-	-	-	-	-
6p21 (HLA-DQB1)	32 771 829	rs6457617	C/T	0.408/ 0.498	1.14E-07	0.69	(0.6–0.79)	0.345/0.463	1.35E-28	0.61	(0.56–0.67)	1.0E-01	2.33E-37	0.62	(0.58–0.67)
**Non MHC loci**															
2p24 (RHOB)	20 548 952	rs342070	C/T	0.293/ 0.226	5.56E-06	1.42	(1.22–1.65)	0.258/0.235	2.61E-02	1.12	(1.01–1.23)	1.9E-01	4.66E-06	1.20	(1.11–1.30)
	20 552 000	rs13021401	T/C	0.289/ 0.225	1.37E-05	1.40	(1.2–1.63)	0.257/0.232	2.47E-02	1.12	(1.01–1.24)	1.3E-01	3.17E-06	1.21	(1.12–1.31)
3p25 (PPARG/TSEN2)	12 468 347	rs9855622	T/C	0.145/ 0.096	1.64E-06	1.66	(1.35–2.05)	0.097/0.109	9.86E-01	1.00	(0.85–1.17)	7.6E-03	1.05E-01	1.11	(0.94–1.17)
	12 234 616	rs310746	C/T	0.121/ 0.08	6.15E-05	1.55	(1.25–1.91)	0.074/0.077	8.69E-02	0.88	(0.77–1.02)	9.6E-01	4.22E-01	1.05	(0.98–1.25)
5q33 (TNIP1)	150 430 429	rs4958881	C/T	0.166/ 0.115	8.26E-06	1.54	(1.28–1.87)	0.151/0.130	4.38E-03	1.21	(1.06–1.38)	3.2E-01	5.79E-06	1.29	(1.17–1.42)
	150 435 925	rs3792783	G/A	0.208/ 0.152	1.14E-05	1.47	(1.24–1.75)	0.198/0.166	2.09E-03	1.21	(1.07–1.36)	6.8E-01	5.73E-07	1.29	(1.20–1.43)
	150 420 290	rs2233287	A/G	0.139/ 0.096	3.71E-05	1.55	(1.26–1.91)	0.121/0.103	4.14E-05	1.26	(1.13–1.40)	6.8E-01	4.68E-09	1.31	(1.15–1.43)
6p16-q16 (ASCC3)	101 444 332	rs9498419	A/G	0.522/ 0.446	7.71E–06	1.37	(1.19–1.57)	0.458/0.475	1.18E-01	0.93	(0.85–1.02)	4.6E-01	3.15E-01	1.04	(0.97–1.11)
	101 445 699	rs6919745	T/C	0.522/ 0.447	8.14E-06	1.37	(1.19–1.57)	0.461/0.478	7.47E-02	0.93	(0.85–1.01)	4.0E-01	3.34E-01	1.04	(0.97–1.11)
7p12-q21 (SEMA3A/HMG17P1)	84 166 013	rs4329228	C/A	0.305/ 0.239	6.66E-06	1.42	(1.22–1.65)	0.249/0.248	5.08E-01	0.97	(0.87–1.07)	3.4E-01	2.05E-01	1.06	(1.04–1.20)
	83 976 940	rs1029541	T/C	0.288/ 0.227	2.37E-05	1.39	(1.2–1.63)	0.223/0.231	7.93E-01	1.01	(0.92–1.12)	9.6E-01	1.50E-02	1.11	(0.97–1.15)
11q25 (OPCML)	132 284 603	rs2725466	G/A	0.403/ 0.328	4.60E-06	1.39	(1.21–1.59)	0.378/0.375	5.71E-01	0.98	(0.89–1.06)	6.2E-01	3.55E-03	1.11	(1.04–1.20)
	132 287 033	rs2725437	C/T	0.404/ 0.335	2.52E-05	1.35	(1.17–1.54)	0.395/0.387	8.33E-01	0.99	(0.91–1.08)	5.4E-01	1.82E-03	1.12	(1.04–1.20)
	132 300 779	rs10894623	T/G	0.317/ 0.256	7.75E-05	1.34	(1.16–1.55)	0.275/0.277	7.67E-01	0.99	(0.90–1.08)	1.4E-01	5.83E-02	1.08	(1.00–1.16)
**Previously reported with P<10^−7^**															
1q22-23 (CD247)	165 687 049	rs2056626	G/T	0.354/0.393	1.70E-02	0.84	(0.73–0.97)	0.36/ 0.4	2.90E-05	0.82	(0.75–0.9)	3.8E-01	1.30E-06	0.83	(0.77–0.89)
2q32 (STAT4)	191 611 003	rs3821236	A/G	0.227/0.203	8.70E-02	1.16	(0.98–1.36)	0.24/ 0.2	2.10E-07	1.33	(1.2–1.49)	8.0E-01	2.09E-07	1.27	(1.16–1.39)
	191 672 878	rs7574865	T/G	0.272/0.219	2.50E-04	1.33	(1.14–1.55)	0.29/ 0.22	1.90E-10	1.40	(1.26–1.56)	9.0E-01	2.26E-13	1.38	(1.27–1.5)
7q32 (TNPO3-IRF5)	128 381 419	rs10488631	C/T	0.124/0.093	2.50E–03	1.39	(1.12–1.72)	0.14/ 0.10	3.49E-05	1.34	(1.17–1.54)	4.5E-01	4.13E-07	1.35	(1.2–1.51)

Outside the MHC region, our GWAS analysis revealed 7 top SNPs (P<10^−5^) that spanned 6 independent genomic regions (Figure 1B and Table S1). Conditional analyses of each of them on *HLA-DQB1* showed no significant drop in the association signals (Table S3). The 6 loci having at least one SNP with a P<10^−5^ were selected for follow-up analysis. Within each locus we selected the SNPs with the strongest (P<10^−4^) association signals to be genotyped in a post-QC replication sample of 1,682 SSc cases and 3,926 controls (Table 1). To this list we added two top SNPs in HLA-DQB1 and the SNP in PSORS1C1. Finally, we further included 4 SNPs at the two known loci (*STAT4* and *TNPO3-IRF5*) and at the newly identified locus (*CD247*) by Radstake et al [18]. Out of a total set of 21 SNPs submitted for replication, 20 passed the quality-control analyses.

**Figure 2:**
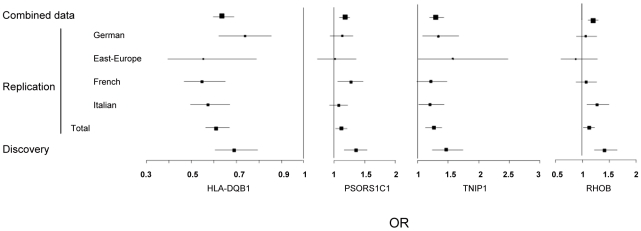
Forest plots showing odds ratios and confidence intervals of the *HLA-DQB1*, *PSORS1C1*, *TNIP1*, and *RHOB* associations in the various populations studied in stage-1 and stage-2 data.

Stratified association analyses in stage 2 data (Table 2), confirmed the strong association for *HLA-DQB1* (rs6457617, P = 1.35×10^−28^) at 6p21.3 and also with the *PSORS1C1* variant (rs3130573, P = 4.98×10^−3^) at 6p21.1. Of the 6 remaining loci selected in stage 1, only 2 were replicated with nominal P<5% and with same direction of effect. They mapped at 2p24 (rs342070, P = 0.026; rs13021401, P = 0.024) and 5q33 (rs3792783, P = 4.14×10^−5^; rs2233287, P = 4.38×10^−3^; rs4958881, P = 2.09×10^−3^). None of the replicated SNPs showed evidence for heterogeneity of effects among the 4 geographical origins (Breslow-day P>0.10). As expected, ORs estimated in the discovery tended to be higher than those obtained in the replication stage data. Afterwards, association signals from joint analyses of the 2 datasets (Table 2) consistently showed highly significant association for *HLA-DQB1* (P = 2.33×10^−37^), *PSORS1C1* (P = 5.70×10^−10^) and *TNIP1* (P = 4.68×10^−9^), and also showed some evidence of association for *RHOB* (P = 3.17×10^−6^). All populations showed same direction of effects (Figure 2). Finally, we also replicated association signals at *IRF5* (P = 3.49×10^−5^; combined-P = 4.13×10^−7^), at STAT4 (P = 1.9×10^−10^; combined-P = 2.26×10^−13^) and at the recently identified new SSc risk locus, *CD247* (P = 2.90×10^−5^; combined-P = 1.30×10^−6^) (Table 2). In our combined data, the locus-specific PAR estimates were 24% for *HLA-DQB1*, 4% for TNIP1, 8% for *PSORS1C1*, 7% for *CD247*, 8% for *STAT4* and 3% with *IFR5/TNPO3*. The combined PAR estimate was 47.4%.

As secondary analyses, we assessed homogeneity of SNP's effect between sub-categories of SSc (cutaneous sub-types and auto-antibodies). Case-only analyses revealed no significant evidence for heterogeneous ORs between cutaneous sub-types of SSc patients for any of the 5 replicated SNPs at 2p24 or 5q33 loci (Table S4A). Indeed, similar association signals were obtained from case-category association analyses (Table S4B). Altogether, the results did not suggest that the association signals in the newly identified 5q33 locus were driven by a specific sub-type of SSc. Conversely, for *HLA-DQB1* and *PSORS1C1* we found evidence of heterogeneity in OR estimates in positive *vs* negative ACA or TOPO auto-antibody SSc patients (Table S4A). Yet, the association signals in each of these sub-types of patients remained strong (Table S4B). These results support the previously reported hypothesis that the magnitude of the *HLA-DQB1* effect on SSc susceptibility may depend on auto-antibody status [11]. The GWAS stage had 78% power to detect loci of the effect sizes observed in the discovery sample for *TNIP1* variants (OR = 1.50) at a significance of P<10^−5^. However, it is widely acknowledged that effect sizes of significant GWAS loci are overestimates of true effects and other genes of lower effect sizes are unlikely to reach stringent significant thresholds.

Our GWAS analysis revealed strong association with *PSORS1C1*, which is ∼1 Mb of *HLA-DQB1*. Notably, *PSORS1C1* is known to be involved in autoimmune response [23]. In the combined data, association with *PSORS1C1* was highly significant (P = 5.70×10^−10^) and remained significant after controlling for the association at HLA-DQB1. Altogether, our results suggest that this region is likely to contain more than one gene playing a role in the pathogenesis of autoimmune disorders [23], [24]. Fine mapping at this locus is warranted to identify causal variants.

**Figure 3:**
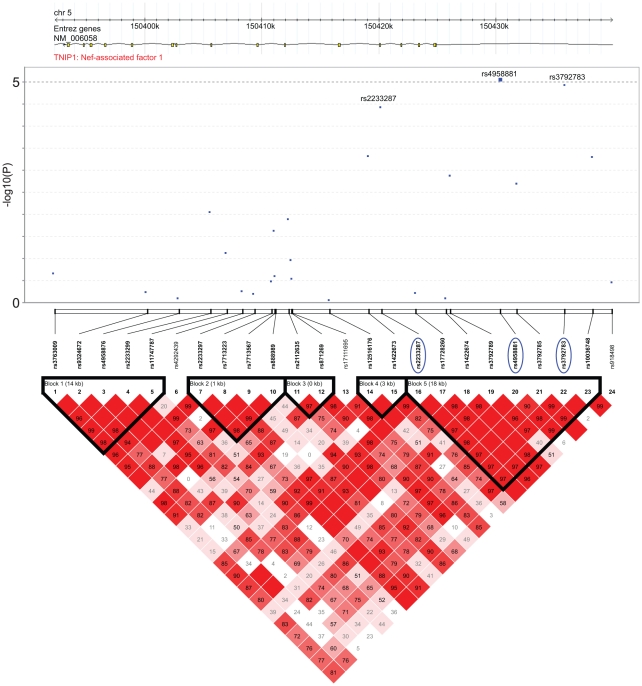
Association and linkage disequilibrium patterns at the TNIP1 gene. (A) Association of SNPs in TNIP1: −log10 P of the logistic regression test for association (y axis) in the GWAS stage of SNPs is plotted against their physical position. The continuous line corresponds to P<10−5, the minimum P value of the top 7 SNPs identified in stage 1. The three SNPs that were followed and replicated in stage 2 are highlighted by a blue circle. Positions are given as NCBI build. (B) Linkage disequilibrim patterns at the TNIP1gene: pairwise LD (D′) are indicated by color gradients: D′≥0.80, red; 0.5≤D′<0.8, pink; 0.2≤D′<0.5, light pink; D′<0.2, white. The 3 SNPs are in strong LD (r2 = 0.57/0.72 between rs3792783 and rs2233287/rs495881). Intron and exon structure of the *TNIP1* gene are taken from the UCSC Genome Browser.

**Figure 4:**
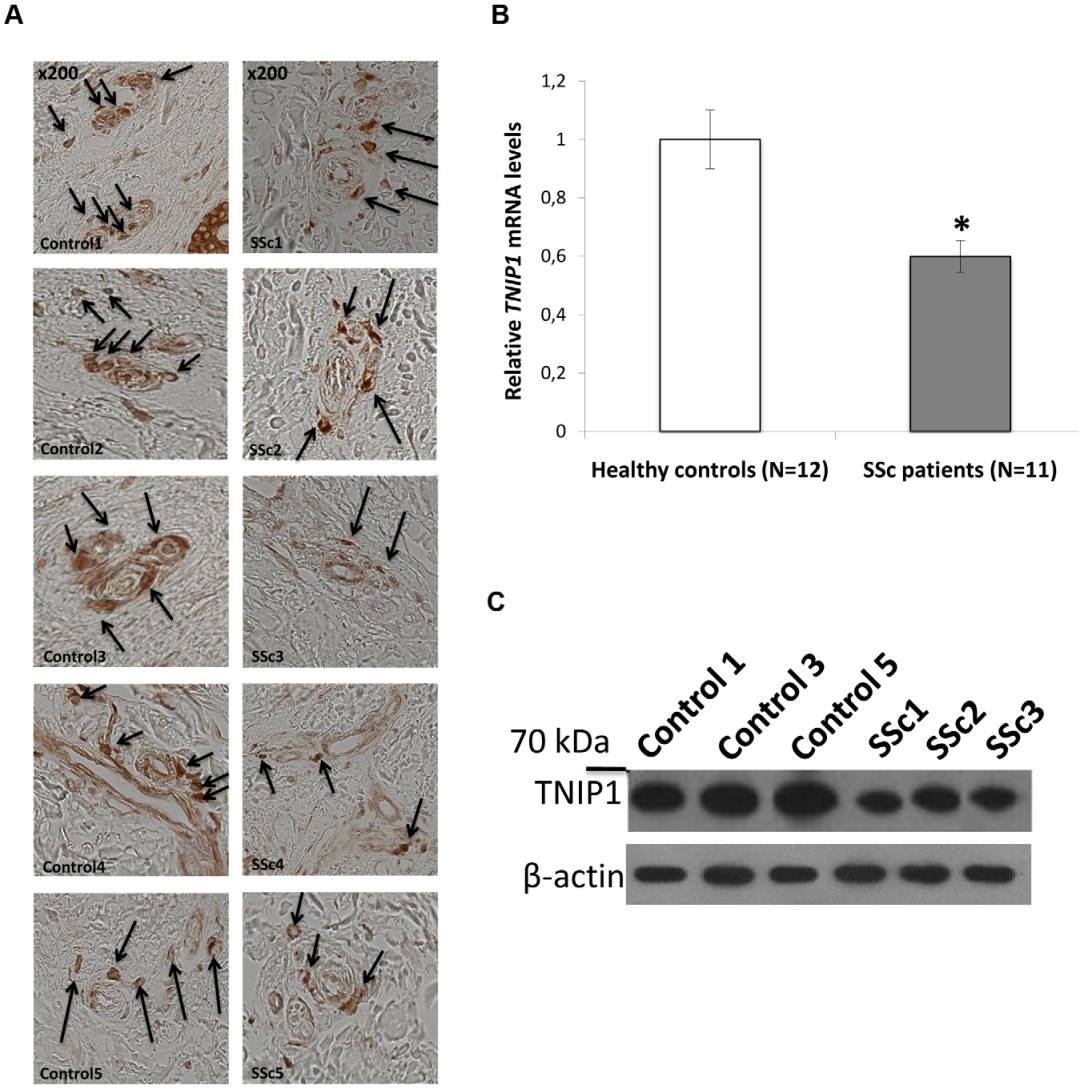
Decreased TNIP1 expression in SSc patients. (A) Expression of the TNIP1 protein was decreased ex vivo in SSc lesional skin tissue compared to controls (arrows indicate TNIP-1 positive cells). Shown are representative sections of the 5 patients and controls included in the analysis. (B) Consistent with these findings, a 1.7-fold decrease of *TNIP1* mRNA levels was observed in dermal fibroblasts from SSc patients (* indicates a *P*-value = 0.001 *vs.* controls). These results were confirmed at the protein level (C).

**Figure 5:**
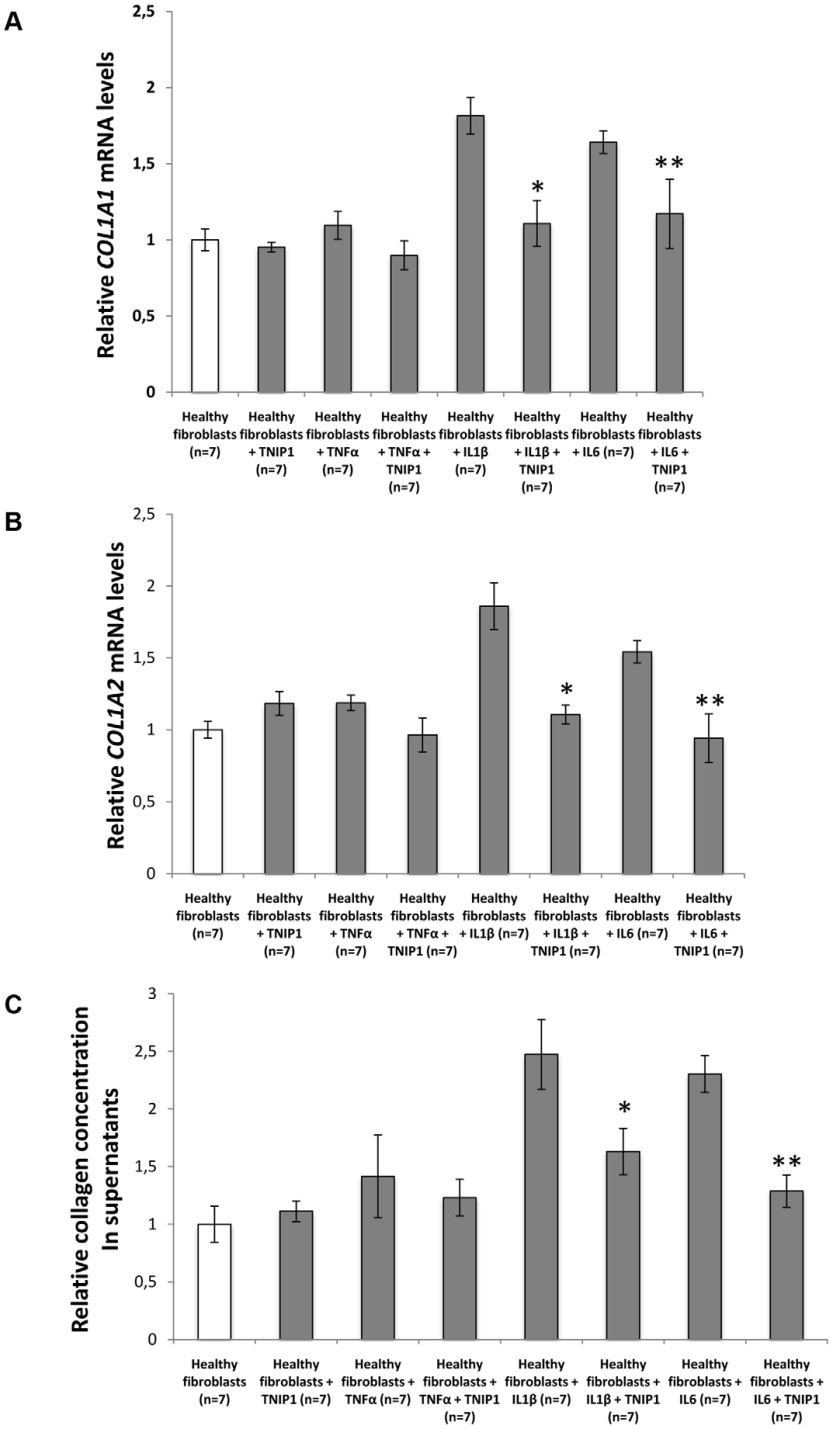
TNIP1 abrogates the profibrotic effects of proinflammatory cytokines on collagen synthesis by healthy fibroblatsts. (A,B) Healthy dermal fibroblasts treated with recombinant Il1β or Il6 and incubated 24 hours with TNIP1 displayed decreased mRNA levels for (A) *COL1A1* (1.6 and 1.4-fold reduction, *P* = 0.01 and 0.03, respectively) and (B) *COL1A2* (1.7 and 1.6-fold reduction, *P* = 0.02 and 0.03, respectively). No significant effect was observed in cells treated with recombinant TNFα. (C) Collagen content in cell culture supernatants treated with Il1β or Il6 was also reduced upon treatment with TNIP1 (1.5 and 1.8-fold reduction, P = 0.02 and 0.03, respectively). * indicates a P<0.05 versus healthy control fibroblasts treated with recombinant IL1β. ** indicates a P<0.05 versus healthy control fibroblasts treated with recombinant IL6.

**Figure 6:**
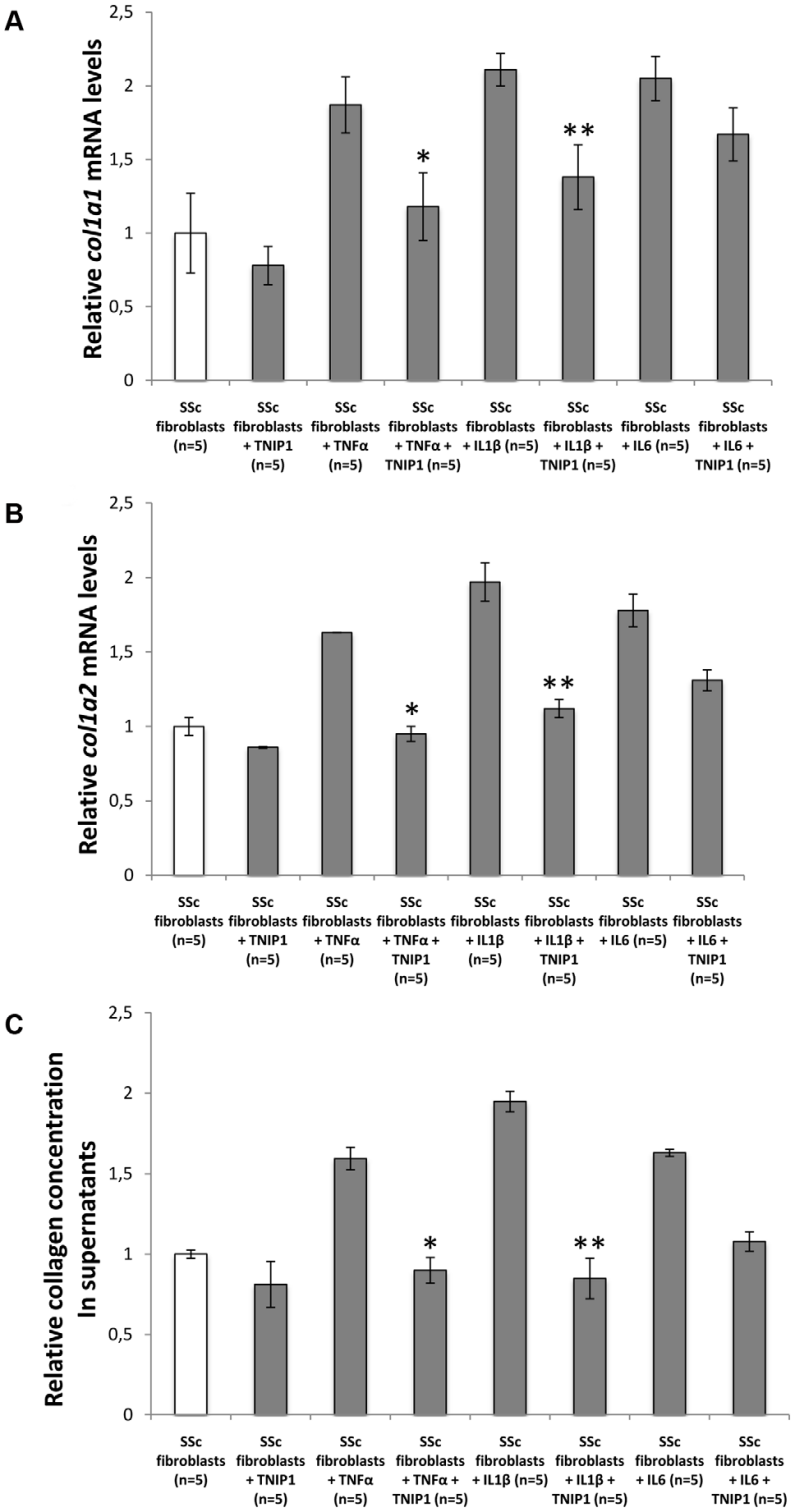
TNIP1 abrogates the profibrotic effects of proinflammatory cytokines on collagen synthesis by SSc fibroblatsts. (A,B) SSc dermal fibroblasts treated with recombinant TNFa or IL1β and incubated 24 hours with TNIP1 displayed decreased mRNA levels for (A) *COL1A1* (1.6 and 1.5-fold reduction, *P* = 0.02 and 0.04, respectively) and (B) *COL1A2* (1.7 and 1.8-fold reduction, *P* = 0.02 and 0.009, respectively). No significant effect was observed in cells treated with recombinant IL6. (C) Collagen content in cell culture supernatants treated with TNFa or IL1β was also reduced upon treatment with TNIP1 (1.8 and 2.3-fold reduction, *P* = 0.04 and 0.03, respectively). * indicates a P<0.05 vs. SSc fibroblasts treated with recombinant TNFa. ** indicates a P<0.05 vs. SSc fibroblasts treated with recombinant IL1 β.

The three strongly associated SNPs at the 5q33 locus are located within the *TNFAIP3* interacting protein 1 (*TNIP1*) gene. *TNIP1* is a very interesting new candidate gene for SSc. The protein encoded by this gene exerts a negative regulation of NF-kappaB via two sequential activities: deubiquitination of Lys63-based chains and synthesis of Lys48-based chains on the TNF receptor-interacting protein and also inhibition of NF-KappaB processing [25]. TNIP1 interacts with A20 (TNFAIP3) to negatively regulate NF-kappaB. Several recent studies have suggested that the activation of some inflammatory factors may upregulate fibrotic mediators through Toll-like receptors (TLRs), thereby contributing to SSc pathogenesis [8]. It has been shown that TLR engagement leads to A20 induction in macrophages and that TNIP1/A20 is essential for the termination of TLR-induced NF-kappaB activity and proinflammatory cytokine production [26]. Although interactions between TNIP1 and A20 are not well known, A20 also acts as a deubiquitinating enzyme, suggesting a molecular link between deubiquitinating activity and the regulation of TLR signals [26]. Therefore, TNIP1 and A20 may play a critical role in the regulation of downstream TLR signals, and this issue will have to be addressed in SSc. Interestingly, variants at *TNIP1* have been shown to be implicated in systemic lupus erythematosus susceptibility [27], [28] and in psoriasis [29]. Furthermore, we have recently reported an association of one *TNFAIP3* variant with SSc [30]. In our stage-1 data, evidence of association at *TNFAIP3* was nominal (lowest P = 0.047) and no pairwise interaction was found (P>0.06) between *TNFAIP3* and *TNIP1* variants. Analysis of the LD structure across the *TNIP1* gene revealed that the 3 strongly associated SNPs belong to the same LD-block (Figure 3). No residual association signals were observed when rs3792783 and each of the other 2 SNPs were paired in conditional analyses. Therefore, any of them, or other variants yet to be identified, could be the causal variant(s). Interestingly, rs3792783 is located upstream from the transcription start site in exon 2 (Figure 3). It is noteworthy that previously reported lupus *TNIP1* variants were located in the same LD-block [27], [28]. Because of the compelling evidence of the potential role of NF-kappaB in autoimmune diseases and our raised new signal association for SSc at *TNIP1* (a negative regulator of this pathway) we performed *ex vivo* and *in vitro* investigations to assess TNIP1 expression in SSc patients and healthy controls. For SSc patients, the results showed a strikingly reduced expression of TNIP1 in skin tissue (Figure 4A), and of both mRNA (Figure 4B) and protein (Figure 4C) synthesis by cultured dermal fibroblasts. Addressing the question of the potential link between the NF-kappaB pathway and the fibrotic propensity that characterizes SSc, we next assessed the influence of pro-inflammatory cytokines and TNIP1 on the synthesis of extra-cellular matrix by dermal fibroblasts in culture. Using cells from the skin of healthy controls (Figure 5) and SSc patients (Figure 6), we showed that recombinant TNIP1 abrogated collagen synthesis induced by inflammatory cytokines both at the mRNA and protein levels. It must be acknowledged that TNIP1 is described as an intra-cellular protein whereas we used recombinant protein added to cell supernatant in these experiments. The observed effects may be related to different hypotheses. TNIP1 has been described as a nuclear shuttling protein and it could have a chaperon-like activity, highly interacting with other protein that could result in engulfment of TNIP1 through interaction with a cell surface protein. Such intra-cellular effects of extra-cellular proteins has been shown also for the S100 family of proteins that have no leader sequence and for clusterin for which it is postulated that the protein could be taken up by interacting with either a yet unidentified receptor or by a mechanism related to their chaperon-like activity [31]. More work is needed to determine which of these hypotheses has to be retained and to investigate more in depth soluble TNIP1 In this first attempt to explore TNIP1 functional disturbances, we could not investigate a relationship between specific TNIP variants and *in vitro* or *in vivo* changes; this will need to be addressed ideally after the identification of the causal variant and using a much larger sample size. Nevertheless, our results raise a potential relationship between inflammation and fibrosis and open a new and highly relevant field of investigation in SSc pathogenesis and in fibrotic disorders.

Our next most associated SNPs at 2p24 are in strong LD (r^2^ = 0.98) and map ∼30 kb from *RHOB*. RHOB is the Ras homolog gene family member B that regulates protein signalling and intracellular protein trafficking. RhoB is essential for activity of farnesyltransferase inhibitors and also statins that are two strong potential future drugs in SSc [9], [32]. To our knowledge, association to RHOB has never been reported so far. The signal for association was weaker at this locus and therefore will need to be confirmed in other samples and more investigations are warranted to assess RHOB implication in this disease.

In conclusion, we have conducted a large genome-wide association study of SSc and identified two new SSc-risk loci, *PSORS1C1* and *TNIP1*. We also confirmed the association of SSc with variants at STAT4, IRF5 and CD247, in the European population. We also found compelling evidence of association to a putative new SSc risk locus on 2p24, close to the *RHOB* gene. None of the newly identified 3 loci have been previously reported associated to SSc. The *TNIP1* variants identified do not have precise functional implications; however, their localization within a regulatory region strongly suggests an impact on transcription of the gene. This is supported by our *ex vivo* and *in vitro* investigations. Altogether, our results are consistent with a reduced inhibition of NF-kappaB, therefore favoring inflammatory/immune responses and potentially contributing to the overproduction of extra-cellular matrix. This raises a new clue for a link between inflammation and SSc that could also be of importance in other fibrotic disorders.

## Material and Methods

### Study populations

Stage-1 included 654 SSc patients and 531 controls recruited through the French GENESYS project [11], [13], [30] and 2,003 controls from the French Three-City (3C) cohort [21], [22]. The stage-2 data included an independent collection of 4,492 samples (pre quality controls) from several University Hospitals in France, Italy, Germany and Eastern Europe. It also included 721 Italian controls recruited through nationwide efforts by HYPERGENE consortium and 481 Illumina HumanHap550 for the KORA S4 study [33], recruited in the city of Augsburg, Southern Germany. In both stage 1 and stage 2 samples, SSc patients fulfilled ACR criteria [34] and were classified in cutaneous subsets according to LeRoy's criteria [19]. Table 1 shows the main characteristics of the post-QC SSc patients and controls.

### Ethics statement

All participants gave written informed consent, and approval was obtained from the relevant local ethical committees.

### Genotyping and quality control analyses

#### Stage 1

French DNA samples from GENESYS and 3C were genotyped at Integragen and at the Centre National de Génotypage (Evry, France), respectively, with Illumina Human610-Quad BeadChip. Data were subjected to standard quality control procedures using tools implemented in PLINK version 1.07 [35]. Markers were removed if they had a genotype-missing rate >0.03 or a minor allele frequency (MAF)<0.05 or a Hardy-Weinberg P< = 10^−5^. Samples were removed on low (<98%) call rate, inconsistencies between reported gender and genotype-determined gender and/or genetic relatedness (identity-by-descent estimate >0.12). Applying these QC filters led to the removal of 791 subjects (56 cases, 735 controls). To detect individuals of non-European ancestry, we computed genome-wide average identity-by-state (IBS) distance with PLINK using a thinned map of 55,193 SNPs. To this end, we removed SNPs in extensive regions of LD (Chr.2, Chr.5, Chr.6, Chr.8, Chr.11) [36], and excluded SNPs if any pair within a 1000-SNPs window had r^2^>0.2. Our data were then merged with genotypes at the same SNPs from 381 unrelated European (CEU), Yoruban (YRI) and Asian (CHB and JPT) samples from the HapMap project. Classical multi-dimensional scaling analysis was applied on the resulting matrix of IBS distances and the first two dimensions were extracted and plotted against each other. The HapMap data were clearly separated into three distinct clusters according to ancestry. Fifty seven of our stage-1 subjects did not cluster within the European group and were excluded from further analyses.

#### Stage 2

Follow-up analysis was conducted for the set of 17 SNPs that were identified in stage I and for 4 SNPs at STAT4, IRF5/TNPO3 and CD247 loci. *De novo* genotyping was performed by a competitive allele-specific PCR system (Kbioscience, Hoddeston, UK) [11], [13], [30]. The additional set of Italian and German control samples were previously genotyped using Illumina 1MQuad or Human610Quad bead chip. One SNP (rs9275224 in HLA) failed genotyping. Accuracy of genotyping was assessed using quality control procedures similar to those applied to our stage 1 data; following the quality control analyses, our stage 2 data consisted of 20 SNPs genotyped in a total of 1,682 cases and 3,926 controls (Table 1).

### Statistical association analyses

Association analysis of the genotype data was conducted with PLINK (v1.07) software [35]. All reported P values are two sided. In stage 1, we applied logistic regression assuming an additive genetic model. The quantile-quantile plot was used to evaluate overall significance of the genome-wide association results and the potential impact of residual population substructure. A conservative genome-wide significance threshold of 0.05/489,918 = 1.02×10^−7^ was used.

Stage 2 association and combined analyses were carried out with the Mantel-Haenszel test to control for differences between geographical groups. A Breslow-Day test was performed to assess the heterogeneity of effects in different populations. In the replication analysis, P values<0.05 and direction of effect as observed in the stage-1 data, were considered to indicate statistical significance.

Secondary statistical analyses were conducted to assess independency of multiple association signals within and between loci and homogeneity of effects between subgroups of SSc patients. Case-only association analyses were conducted using the three main clinical variables (Table 1). The LD structure of the identified loci was analyzed using Haploview 4.1 [37] and LD blocks delimited using the D′-based confidence interval method [38]. The locus-specific Population attributable risk (PAR) was calculated for each of the 6 replicated loci (HLA-DQB1, TNIP1, PSORS1C1, STAT4, IFR5/TNPO3 and CD247) according to the following formula: PAR = RAF×(OR-1)/(RAF×(OR-1)+1), where RAF is the frequency of the associated allele in the controls, and OR is the odds ratio associated with the risk allele. The combined PAR was computed as 1−P_j_(1−PAR_j_).

### Histologic and cytologic investigations

Fibroblast cultures were prepared by outgrowth cultures from lesional skin biopsy specimens of eleven SSc patients and from twelve healthy controls matched for age and sex. The median age of SSc patients was 49 years old (range: 22–67 years) and their median disease duration was 7 years (range: 1–17 years); seven had the limited cutaneous subset and four the diffuse. Immunohistochemistry was performed on paraffin-embedded skin sections from 5 SSc patients and 5 controls using mouse anti-human TNIP1 antibodies (eBioscience, Frankfurt, Germany). Total RNA, issued from cultured dermal fibroblasts, isolation and reverse transcription into complementary DNA were performed as previously described [39]. Gene expression was quantified by SYBR Green real-time PCR, with a specific primer pair available upon request. Protein assessment was performed on western blots, as previously described [40] using mouse anti-human TNIP1 antibodies (eBioscience, CA, USA). In selected experiments, dermal fibroblasts from healthy control subjects and patients with SSc were treated for 24 hours with recombinant TNIP1 (2 µg/ml, Abnova, Tapei City, Taiwan) in the presence or not of the following proinflammatory cytokines: TNFα (20 ng/ml, R&D systems, Abingdon, UK), IL1β (1 µg/ml, Immunotools, Friesoythe, Germany) or IL6 (1 µg/ml, Immunotools). mRNA levels of human α1(I) and α2(I) procollagen were quantified by quantitative real-time PCR, specific primers are available upon request. The collagen content in cell culture supernatants was analyzed with the SirCol collagen assay (Biocolor, Belfast, UK) [41]. Comparisons were performed using Student's T test.

## Supporting Information

Table S1 GWAS results for the most associated (P<10^−4^) SNPs.
(DOC)

Table S2 Results of conditional logistic regression analysis for 28 SNPs (P<10^−3^) in the MHC region.
(DOC)

Table S3 Results of Conditional logistic regression analysis for top 7 SNPs outside the MHC region in GWAS data.
(DOC)

Table S4 Association results in the combined (stage-1 and stage-2) data for the replicated SNPs by sub-type of SSc patients.
(DOC)
