# Supplementary material for: Genome-Wide Scan Identifies TNIP1, PSORS1C1, and RHOB as Novel Risk Loci for Systemic Sclerosis
Source: PLoS Genet. 2011 Jul 7;7(7):e1002091. doi: 10.1371/journal.pgen.1002091 (PMC3131285; doi:10.1371/journal.pgen.1002091)
Supplement: Table S1 — GWAS results for the most associated (P<10−4) SNPs. (DOC) [file pgen.1002091.s001.doc]

**Table S1: GWAS results for the most associated (P<10^-4^**) SNPs

| **Chr. (gene)** | **Pos. (bp)** | **SNP** | **Minor/ Major** | **N Cases/ Controls** | **MAF Cases/ Controls** | **P** | **OR** | **(95% CI)** |
| --- | --- | --- | --- | --- | --- | --- | --- | --- |
|  |  |  |  |  |  |  |  |  |
| 1 | 22 858 990 | rs631090 | C/T | 564/ 1776 | 0.098/ 0.063 | 8.80E-05 | 1.61 | (1.27-2.05) |
| 1 | 36 846 938 | rs4653210 | G/T | 564/ 1774 | 0.098/ 0.147 | 2.60E-05 | 0.63 | (0.5-0.78) |
| 1 | 38 579 217 | rs6679637 | A/G | 564/ 1776 | 0.081/ 0.125 | 6.29E-05 | 0.62 | (0.49-0.78) |
| 1 | 237 572 700 | rs10925871 | A/G | 564/ 1775 | 0.209/ 0.157 | 6.38E-05 | 1.41 | (1.19-1.67) |
| 2 | 10 631 383 | rs4668690 | A/G | 564/ 1774 | 0.097/ 0.061 | 8.05E-05 | 1.61 | (1.27-2.04) |
| 2 | 10 768 380 | rs7422405 | A/G | 564/ 1774 | 0.406/ 0.475 | 4.55E-05 | 0.75 | (0.65-0.86) |
| 2q24 (RHOB) | 20 548 952 | rs342070 | C/T | 564/ 1776 | 0.293/ 0.226 | **5.56E-06** | 1.42 | (1.22-1.65) |
| 2q24 (RHOB) | 20 552 000 | rs13021401 | T/C | 564/ 1772 | 0.289/ 0.225 | 1.37E-05 | 1.4 | (1.2-1.63) |
| 2 | 71 544 368 | rs11692280 | A/G | 564/ 1743 | 0.166/ 0.228 | 1.08E-05 | 0.67 | (0.56-0.8) |
| 3 | 10 252 866 | rs11706450 | T/C | 564/ 1776 | 0.426/ 0.491 | 7.86E-05 | 0.75 | (0.66-0.87) |
| 3 | 12 234 616 | rs310746 | C/T | 564/ 1775 | 0.121/ 0.08 | 6.15E-05 | 1.55 | (1.25-1.91) |
| 3 | 12 468 347 | rs9855622 | T/C | 564/ 1774 | 0.145/ 0.096 | **1.64E-06** | 1.66 | (1.35-2.05) |
| 3 | 29 572 795 | rs35883 | A/G | 564/ 1775 | 0.498/ 0.431 | 8.60E-05 | 1.31 | (1.14-1.5) |
| 3 | 29 595 608 | rs13323338 | C/T | 564/ 1772 | 0.383/ 0.314 | 2.06E-05 | 1.35 | (1.18-1.55) |
| 3 | 46 619 913 | rs6799581 | G/T | 564/ 1775 | 0.244/ 0.307 | 5.35E-05 | 0.73 | (0.62-0.85) |
| 3 | 118 635 074 | rs4128236 | T/C | 562/ 1775 | 0.347/ 0.284 | 6.60E-05 | 1.34 | (1.16-1.55) |
| 4 | 39 998 408 | rs6832151 | G/T | 560/ 1774 | 0.347/ 0.284 | 6.11E-05 | 1.34 | (1.16-1.55) |
| 4 | 95 443 835 | rs17021463 | T/G | 564/ 1776 | 0.384/ 0.451 | 9.48E-05 | 0.76 | (0.66-0.87) |
| 4 | 156 293 524 | rs13138293 | G/T | 564/ 1776 | 0.348/ 0.285 | 5.47E-05 | 1.34 | (1.16-1.55) |
| 4 | 156 300 178 | rs2880417 | G/A | 564/ 1767 | 0.328/ 0.267 | 9.57E-05 | 1.33 | (1.15-1.54) |
| 5 | 3 504 906 | rs32723 | T/G | 564/ 1774 | 0.35/ 0.418 | 6.19E-05 | 0.75 | (0.66-0.87) |
| 5 | 19 955 058 | rs1911856 | T/C | 564/ 1776 | 0.075/ 0.042 | 1.04E-05 | 1.88 | (1.42-2.48) |
| 5 | 19 959 548 | rs12655266 | A/G | 564/ 1770 | 0.09/ 0.055 | 3.08E-05 | 1.71 | (1.33-2.2) |
| 5 | 19 962 282 | rs2202798 | T/C | 564/ 1773 | 0.096/ 0.059 | 2.60E-05 | 1.69 | (1.32-2.16) |
| 5 | 62 129 790 | rs7708428 | G/A | 563/ 1774 | 0.379/ 0.45 | 4.54E-05 | 0.75 | (0.66-0.86) |
| 5q33 (TNIP1) | 150 420 290 | rs2233287 | A/G | 564/ 1769 | 0.139/ 0.096 | 3.71E-05 | 1.55 | (1.26-1.91) |
| 5q33 (TNIP1) | 150 430 429 | rs4958881 | C/T | 563/ 1775 | 0.166/ 0.115 | **8.26E-06** | 1.54 | (1.28-1.87) |
| 5q33 (TNIP1) | 150 435 925 | rs3792783 | G/A | 564/ 1773 | 0.208/ 0.152 | 1.14E-05 | 1.47 | (1.24-1.75) |
| 5 | 168 225 317 | rs2938769 | T/C | 564/ 1775 | 0.365/ 0.435 | 2.86E-05 | 0.74 | (0.64-0.85) |
| 6p21(PSORS1C1) | 31 214 247 | rs3130573 | G/A | 562/ 1774 | 0.391/ 0.321 | 1.86E-05 | 1.36 | (1.18-1.56) |
| 6p21 (BTNL2) | 32 374 468 | rs28366174 | C/T | 564/ 1772 | 0.06/ 0.1 | 7.81E-05 | 0.58 | (0.44-0.76) |
| 6p21 (HLA-DQB1) | 32 767 856 | rs9275224 | A/G | 563/ 1772 | 0.405/ 0.496 | **9.18E-08** | 0.69 | (0.6-0.79) |
|  | 32 768 921 | rs9275245 | A/G | 564/ 1759 | 0.406/ 0.496 | **1.39E-07** | 0.69 | (0.6-0.79) |
|  | 32 771 829 | rs6457617 | C/T | 564/ 1775 | 0.41/ 0.5 | **1.14E-07** | 0.69 | (0.6-0.79) |
|  | 32 777 978 | rs2858308 | T/G | 564/ 1776 | 0.081/ 0.125 | 4.76E-05 | 0.61 | (0.48-0.77) |
|  | 32 778 934 | rs2856705 | T/C | 564/ 1776 | 0.081/ 0.125 | 4.76E-05 | 0.61 | (0.48-0.77) |
|  | 32 779 081 | rs13192471 | C/T | 564/ 1769 | 0.19/ 0.136 | 1.81E-05 | 1.47 | (1.23-1.75) |
| 6 | 101 444 332 | rs9498419 | A/G | 563/ 1758 | 0.522/ 0.446 | **7.71E-06** | 1.37 | (1.19-1.57) |
| 6 | 101 445 699 | rs6919745 | T/C | 564/ 1776 | 0.522/ 0.447 | **8.14E-06** | 1.37 | (1.19-1.57) |
| 6 | 101 446 121 | rs7771570 | C/T | 563/ 1771 | 0.536/ 0.463 | 1.82E-05 | 1.35 | (1.18-1.55) |
| 7 | 25 877 558 | rs10272701 | T/C | 564/ 1774 | 0.215/ 0.164 | 9.22E-05 | 1.39 | (1.18-1.64) |
| 7 | 70 542 497 | rs4585627 | T/C | 564/ 1775 | 0.353/ 0.288 | 3.42E-05 | 1.36 | (1.18-1.57) |
| 7 | 81 843 406 | rs1544461 | A/G | 564/ 1776 | 0.45/ 0.38 | 3.64E-05 | 1.33 | (1.16-1.53) |
| 7 | 83 866 854 | rs1228870 | T/G | 564/ 1764 | 0.275/ 0.218 | 7.62E-05 | 1.37 | (1.17-1.6) |
| 7 | 83 924 438 | rs1228966 | A/G | 564/ 1776 | 0.277/ 0.22 | 8.77E-05 | 1.37 | (1.17-1.6) |
| 7 | 83 975 558 | rs757747 | T/C | 564/ 1770 | 0.285/ 0.226 | 5.57E-05 | 1.37 | (1.18-1.6) |
| 7 | 83 976 940 | rs1029541 | T/C | 564/ 1775 | 0.288/ 0.227 | 2.37E-05 | 1.39 | (1.2-1.63) |
| 7 | 84 166 013 | rs4329228 | C/A | 564/ 1775 | 0.305/ 0.239 | **6.66E-06** | 1.42 | (1.22-1.65) |
| 7 | 116 005 911 | rs2402091 | A/G | 564/ 1773 | 0.095/ 0.142 | 5.34E-05 | 0.63 | (0.51-0.79) |
| 7 | 122 314 434 | rs2501439 | G/A | 564/ 1776 | 0.379/ 0.445 | 9.29E-05 | 0.76 | (0.66-0.87) |
| 8 | 42 776 255 | rs6474421 | G/A | 563/ 1723 | 0.046/ 0.085 | 2.80E-05 | 0.52 | (0.39-0.71) |
| 8 | 124 583 581 | rs3739284 | C/T | 564/ 1775 | 0.203/ 0.261 | 9.31E-05 | 0.72 | (0.61-0.85) |
| 8 | 131 230 753 | rs6470805 | G/A | 564/ 1772 | 0.302/ 0.366 | 8.60E-05 | 0.75 | (0.65-0.86) |
| 8 | 131 351 008 | rs7839523 | G/T | 564/ 1776 | 0.468/ 0.399 | 4.86E-05 | 1.32 | (1.16-1.51) |
| 8 | 131 389 004 | rs7817803 | A/C | 557/ 1774 | 0.464/ 0.394 | 4.73E-05 | 1.32 | (1.16-1.51) |
| 8 | 131 399 019 | rs3057 | C/T | 563/ 1776 | 0.47/ 0.397 | 2.09E-05 | 1.34 | (1.17-1.53) |
| 9 | 11 699 025 | rs443042 | G/A | 564/ 1776 | 0.309/ 0.375 | 6.35E-05 | 0.75 | (0.65-0.86) |
| 9 | 11 700 328 | rs10756265 | A/G | 563/ 1776 | 0.288/ 0.354 | 4.80E-05 | 0.74 | (0.64-0.85) |
| 9 | 94 884 973 | rs9696357 | T/C | 564/ 1771 | 0.145/ 0.198 | 9.08E-05 | 0.69 | (0.57-0.83) |
| 9 | 99 522 324 | rs2668797 | A/G | 561/ 1772 | 0.37/ 0.441 | 2.90E-05 | 0.74 | (0.65-0.86) |
| 9 | 99 535 981 | rs2805815 | A/G | 564/ 1776 | 0.373/ 0.442 | 6.08E-05 | 0.76 | (0.66-0.87) |
| 9 | 99 539 027 | rs2805790 | A/G | 564/ 1775 | 0.372/ 0.441 | 5.20E-05 | 0.75 | (0.66-0.86) |
| 9 | 99 545 651 | rs2808699 | A/C | 563/ 1774 | 0.372/ 0.442 | 4.41E-05 | 0.75 | (0.65-0.86) |
| 9 | 137 692 055 | rs541131 | G/A | 564/ 1775 | 0.411/ 0.339 | 1.63E-05 | 1.36 | (1.18-1.56) |
| 10 | 43 768 026 | rs1254860 | C/T | 564/ 1774 | 0.138/ 0.098 | 9.69E-05 | 1.51 | (1.23-1.86) |
| 11 | 46 137 269 | rs7128538 | A/G | 564/ 1775 | 0.509/ 0.436 | 2.55E-05 | 1.33 | (1.17-1.52) |
| 11 | 132 280 051 | rs11223273 | T/C | 564/ 1776 | 0.302/ 0.244 | 9.91E-05 | 1.34 | (1.16-1.56) |
| 11 | 132 284 603 | rs2725466 | G/A | 564/ 1772 | 0.403/ 0.328 | **4.60E-06** | 1.39 | (1.21-1.59) |
| 11 | 132 287 033 | rs2725437 | C/T | 564/ 1775 | 0.404/ 0.335 | 2.52E-05 | 1.35 | (1.17-1.54) |
| 11 | 132 300 779 | rs10894623 | T/G | 564/ 1776 | 0.317/ 0.256 | 7.75E-05 | 1.34 | (1.16-1.55) |
| 12 | 130 766 115 | rs10794423 | C/T | 564/ 1776 | 0.381/ 0.449 | 6.32E-05 | 0.75 | (0.66-0.87) |
| 13 | 33 608 780 | rs7335534 | G/A | 564/ 1772 | 0.38/ 0.448 | 9.70E-05 | 0.76 | (0.67-0.87) |
| 13 | 33 614 493 | rs11147544 | G/A | 562/ 1754 | 0.321/ 0.387 | 8.26E-05 | 0.75 | (0.65-0.87) |
| 14 | 32 653 876 | rs1299512 | G/A | 564/ 1774 | 0.259/ 0.203 | 8.34E-05 | 1.37 | (1.17-1.6) |
| 14 | 82 294 856 | rs1036570 | A/G | 564/ 1768 | 0.277/ 0.342 | 4.88E-05 | 0.73 | (0.63-0.85) |
| 15 | 65 212 087 | rs12102171 | T/C | 562/ 1772 | 0.164/ 0.221 | 3.99E-05 | 0.69 | (0.58-0.82) |
| 15 | 65 215 068 | rs4147358 | A/C | 564/ 1770 | 0.213/ 0.278 | 1.65E-05 | 0.7 | (0.6-0.83) |
| 16 | 48 275 072 | rs1990629 | G/A | 564/ 1775 | 0.098/ 0.145 | 7.56E-05 | 0.64 | (0.52-0.8) |
| 16 | 48 277 741 | rs1477020 | T/C | 564/ 1773 | 0.09/ 0.139 | 2.49E-05 | 0.61 | (0.49-0.77) |
| 16 | 63 863 763 | rs1423773 | A/G | 561/ 1770 | 0.321/ 0.387 | 6.57E-05 | 0.75 | (0.65-0.86) |
| 17 | 30 050 804 | rs4795032 | T/C | 564/ 1776 | 0.384/ 0.315 | 2.93E-05 | 1.34 | (1.17-1.54) |
| 17 | 30 102 223 | rs887081 | T/G | 563/ 1776 | 0.099/ 0.148 | 5.17E-05 | 0.64 | (0.52-0.79) |
| 17 | 35 346 239 | rs8079416 | C/T | 564/ 1775 | 0.406/ 0.48 | 1.11E-05 | 0.73 | (0.64-0.84) |
| 18 | 1 992 665 | rs1940646 | A/G | 562/ 1775 | 0.379/ 0.316 | 9.06E-05 | 1.32 | (1.15-1.52) |
| 18 | 58 834 621 | rs2877745 | T/C | 564/ 1776 | 0.103/ 0.066 | 3.40E-05 | 1.65 | (1.3-2.1) |
| 18 | 70 337 384 | rs2241508 | G/A | 564/ 1755 | 0.457/ 0.386 | 1.80E-05 | 1.35 | (1.18-1.55) |
| 19 | 11 278 200 | rs322151 | T/C | 564/ 1775 | 0.285/ 0.227 | 7.79E-05 | 1.37 | (1.17-1.59) |
| 21 | 18 396 486 | rs2248200 | C/T | 563/ 1773 | 0.513/ 0.446 | 8.85E-05 | 1.31 | (1.15-1.5) |
| 21 | 18 396 648 | rs1688165 | A/G | 564/ 1774 | 0.513/ 0.446 | 8.27E-05 | 1.31 | (1.15-1.5) |
| 21 | 28 407 230 | rs2831511 | T/C | 564/ 1776 | 0.364/ 0.429 | 9.71E-05 | 0.76 | (0.66-0.87) |
| 22 | 17 257 787 | rs2543958 | G/T | 564/ 1750 | 0.149/ 0.105 | 7.46E-05 | 1.49 | (1.22-1.81) |

**Supporting Table 3 (S3):** Results of Conditional logistic regression analysis for top 7 SNPs outside the MHC region in GWAS data

|  |  |  |  | **Controlling on** | | | | | | | |
| --- | --- | --- | --- | --- | --- | --- | --- | --- | --- | --- | --- |
|  |  |  |  | **rs6457617** | |  | **rs3130573** | |  | **rs6457617 & rs3130573** | |
| **Chr.** | **SNP** | **Pos** | **A1** | **OR** | **P** |  | **OR** | **P** |  | **OR** | **P** |
| 2 | rs342070 | 20 548 952 | C | 1.41 | 1.04E-05 |  | 1.41 | 8.74E-06 |  | 1.40 | 1.70E-05 |
| 3 | rs9855622 | 12 468 347 | T | 1.64 | 3.48E-06 |  | 1.67 | 1.39E-06 |  | 1.65 | 2.97E-06 |
| 5 | rs4958881 | 150 430 429 | C | 1.54 | 1.19E-05 |  | 1.55 | 7.19E-06 |  | 1.54 | 1.18E-05 |
| 6 | rs9498419 | 101 444 332 | A | 1.37 | 7.76E-06 |  | 1.37 | 7.34E-06 |  | 1.37 | 7.36E-06 |
| 6 | rs6919745 | 101 445 699 | T | 1.37 | 8.21E-06 |  | 1.37 | 7.86E-06 |  | 1.37 | 7.90E-06 |
| 7 | rs4329228 | 84 166 013 | C | 1.41 | 1.08E-05 |  | 1.42 | 7.64E-06 |  | 1.41 | 1.25E-05 |
| 11 | rs2725466 | 132 284 603 | G | 1.38 | 6.19E-06 |  | 1.39 | 3.78E-06 |  | 1.39 | 5.13E-06 |

**Supporting Table 4 (S4): Association results in the combined data for the replicated SNPs by sub-type of SSc patients**

|  | **A. Case -Only analyses. Homogeneity of ORs by case category** | | | | | | | | | | |  |  |  |  |  |  |  |  |  |
| --- | --- | --- | --- | --- | --- | --- | --- | --- | --- | --- | --- | --- | --- | --- | --- | --- | --- | --- | --- | --- |
|  |  |  |  | **^£^Dc vs Lc** | |  | **^$^ACA+ vs ACA-** | |  | **^*^TOPO+ vs TOPO-** | |  |  |  |  |  |  |  |  |  |
| **Chr.-gene)** | **SNP** | **BP** | **Min.** | **P** | **OR** |  | **P** | **OR** |  | **P** | **OR** |  |  |  |  |  |  |  |  |  |
| 6p21(PSORS1C1) | rs3130573 | 31 214 247 | G | 7.3E-01 | 1.02 |  | **2.1E-02** | 0.86 |  | 2.9E-01 | 1.08 |  |  |  |  |  |  |  |  |  |
| 6p21 (HLA-DQB1) | rs6457617 | 32 771 829 | C | 7.8E-01 | 1.02 |  | **2.1E-04** | 0.78 |  | **2.6E-02** | 0.85 |  |  |  |  |  |  |  |  |  |
|  |  |  |  |  |  |  |  |  |  |  |  |  |  |  |  |  |  |  |  |  |
| 2 (RHOB) | rs342070 | 20 548 952 | C | 1.6E-01 | 0.90 |  | 9.4E-01 | 0.99 |  | 4.7E-01 | 0.94 |  |  |  |  |  |  |  |  |  |
|  | rs13021401 | 20 552 000 | T | 2.0E-01 | 0.91 |  | 6.5E-01 | 0.97 |  | 6.3E-01 | 0.96 |  |  |  |  |  |  |  |  |  |
| 5 (TNIP1) | rs4958881 | 150 430 429 | A | 4.6E-01 | 1.07 |  | 1.6E-01 | 0.88 |  | 1.6E-01 | 1.14 |  |  |  |  |  |  |  |  |  |
|  | rs3792783 | 150 435 925 | C | 7.5E-01 | 1.03 |  | 8.2E-01 | 0.98 |  | 3.1E-01 | 1.09 |  |  |  |  |  |  |  |  |  |
|  | rs2233287 | 150 420 290 | G | 5.7E-01 | 1.06 |  | 2.3E-01 | 0.89 |  | 4.0E-01 | 1.09 |  |  |  |  |  |  |  |  |  |
|  |  |  |  |  |  |  |  |  |  |  |  |  |  |  |  |  |  |  |  |  |
|  |  |  |  |  |  |  |  |  |  |  |  |  |  |  |  |  |  |  |  |  |
|  | **B. Case category vs Controls analyses. Evidence of association by case category** | | | | | | | | | | |  |  |  |  |  |  |  |  |  |
|  |  |  |  | **Dc** | |  | **Lc** | |  | **ACA+** | |  | **ACA-** | |  | **TOPO+** | |  | **TOPO-** | |
| **Chr.-gene)** | **SNP** | **BP** | **Min.** | **P** | **OR** |  | **P** | **OR** |  | **P** | **OR** |  | **P** | **OR** |  | **P** | **OR** |  | **P** | **OR** |
| 6p21(PSORS1C1) | rs3130573 | 31 214 247 | G | 2.6E-03 | 1.20 |  | 2.6E-03 | 1.14 |  | 2.3E-01 | 1,07 |  | 4.2E-06 | 1,25 |  | 1.2E-03 | 1.23 |  | 2.6E-03 | 1.15 |
| 6p21 (HLA-DQB1) | rs6457617 | 32 771 829 | C | 2.9E-12 | 0.65 |  | 3.2E-20 | 0.66 |  | 5.7E-27 | 0,54 |  | 5.3E-14 | 0,70 |  | 3.9E-18 | 0,57 |  | 8.7E-20 | 0,66 |
|  |  |  |  |  |  |  |  |  |  |  |  |  |  |  |  |  |  |  |  |  |
| 2 (RHOB) | rs342070 | 20 548 952 | C | 1.5E-01 | 1.11 |  | 1.1E-05 | 1.24 |  | 1.9E-03 | 1.21 |  | 1.1E-03 | 1.19 |  | 5.5E-02 | 1.15 |  | 5.1E-05 | 1.22 |
|  | rs13021401 | 20 552 000 | T | 1.5E-01 | 1.11 |  | 2.7E-05 | 1.23 |  | 5.7E-03 | 1.19 |  | 7.2E-04 | 1.20 |  | 3.7E-02 | 1.16 |  | 1.1E-04 | 1.21 |
| 5 (TNIP1) | rs4958881 | 150 430 429 | A | 1.5E-03 | 1.33 |  | 5.4E-03 | 1.20 |  | 5.6E-02 | 1.18 |  | 2.3E-05 | 1.35 |  | 1.4E-03 | 1.35 |  | 1.3E-03 | 1.24 |
|  | rs3792783 | 150 435 925 | C | 6.3E-04 | 1.33 |  | 3.9E-03 | 1.19 |  | 5.2E-02 | 1.16 |  | 8.1E-06 | 1.34 |  | 1.1E-04 | 1.39 |  | 1.5E-03 | 1.22 |
|  | rs2233287 | 150 420 290 | G | 7.6E-05 | 1.35 |  | 5.7E-05 | 1.25 |  | 3.0E-04 | 1.28 |  | 1.9E-06 | 1.33 |  | 1.8E-05 | 1.39 |  | 1.3E-05 | 1.28 |

£ Diffuse cutaneous (Dc) vs Limited cutaneous (Lc) forms; $anticentromere antibodies positive (ACA+) vs negative (ACA-); *anti-topoisomerase I positive
