# Supplementary material for: Genome-Wide Scan Identifies TNIP1, PSORS1C1, and RHOB as Novel Risk Loci for Systemic Sclerosis
Source: PLoS Genet. 2011 Jul 7;7(7):e1002091. doi: 10.1371/journal.pgen.1002091 (PMC3131285; doi:10.1371/journal.pgen.1002091)
Supplement: Table S3 — Results of Conditional logistic regression analysis for top 7 SNPs outside the MHC region in GWAS data. (DOC) [file pgen.1002091.s003.doc]

**Table S3:** Results of Conditional logistic regression analysis for top 7 SNPs outside the MHC region in GWAS data

|  |  |  |  | **Controlling on** | | | | | | | |
| --- | --- | --- | --- | --- | --- | --- | --- | --- | --- | --- | --- |
|  |  |  |  | **rs6457617** | |  | **rs3130573** | |  | **rs6457617 & rs3130573** | |
| **Chr.** | **SNP** | **Pos** | **A1** | **OR** | **P** |  | **OR** | **P** |  | **OR** | **P** |
| 2 | rs342070 | 20 548 952 | C | 1.41 | 1.04E-05 |  | 1.41 | 8.74E-06 |  | 1.40 | 1.70E-05 |
| 3 | rs9855622 | 12 468 347 | T | 1.64 | 3.48E-06 |  | 1.67 | 1.39E-06 |  | 1.65 | 2.97E-06 |
| 5 | rs4958881 | 150 430 429 | C | 1.54 | 1.19E-05 |  | 1.55 | 7.19E-06 |  | 1.54 | 1.18E-05 |
| 6 | rs9498419 | 101 444 332 | A | 1.37 | 7.76E-06 |  | 1.37 | 7.34E-06 |  | 1.37 | 7.36E-06 |
| 6 | rs6919745 | 101 445 699 | T | 1.37 | 8.21E-06 |  | 1.37 | 7.86E-06 |  | 1.37 | 7.90E-06 |
| 7 | rs4329228 | 84 166 013 | C | 1.41 | 1.08E-05 |  | 1.42 | 7.64E-06 |  | 1.41 | 1.25E-05 |
| 11 | rs2725466 | 132 284 603 | G | 1.38 | 6.19E-06 |  | 1.39 | 3.78E-06 |  | 1.39 | 5.13E-06 |
