# Supplementary material for: Genome-Wide Scan Identifies TNIP1, PSORS1C1, and RHOB as Novel Risk Loci for Systemic Sclerosis
Source: PLoS Genet. 2011 Jul 7;7(7):e1002091. doi: 10.1371/journal.pgen.1002091 (PMC3131285; doi:10.1371/journal.pgen.1002091)
Supplement: Table S4 — Association results in the combined (stage-1 and stage-2) data for the replicated SNPs by sub-type of SSc patients. (DOC) [file pgen.1002091.s004.doc]

**Table S4: Association results in the combined data for the replicated SNPs by sub-type of SSc patients**

|  | **A. Case -Only analyses. Homogeneity of ORs by case category** | | | | | | | | | | |  |  |  |  |  |  |  |  |  |
| --- | --- | --- | --- | --- | --- | --- | --- | --- | --- | --- | --- | --- | --- | --- | --- | --- | --- | --- | --- | --- |
|  |  |  |  | **^£^Dc vs Lc** | |  | **^$^ACA+ vs ACA-** | |  | **^*^TOPO+ vs TOPO-** | |  |  |  |  |  |  |  |  |  |
| **Chr.-gene)** | **SNP** | **BP** | **Min.** | **P** | **OR** |  | **P** | **OR** |  | **P** | **OR** |  |  |  |  |  |  |  |  |  |
| 6p21(PSORS1C1) | rs3130573 | 31 214 247 | G | 7.3E-01 | 1.02 |  | **2.1E-02** | 0.86 |  | 2.9E-01 | 1.08 |  |  |  |  |  |  |  |  |  |
| 6p21 (HLA-DQB1) | rs6457617 | 32 771 829 | C | 7.8E-01 | 1.02 |  | **2.1E-04** | 0.78 |  | **2.6E-02** | 0.85 |  |  |  |  |  |  |  |  |  |
|  |  |  |  |  |  |  |  |  |  |  |  |  |  |  |  |  |  |  |  |  |
| 2 (RHOB) | rs342070 | 20 548 952 | C | 1.6E-01 | 0.90 |  | 9.4E-01 | 0.99 |  | 4.7E-01 | 0.94 |  |  |  |  |  |  |  |  |  |
|  | rs13021401 | 20 552 000 | T | 2.0E-01 | 0.91 |  | 6.5E-01 | 0.97 |  | 6.3E-01 | 0.96 |  |  |  |  |  |  |  |  |  |
| 5 (TNIP1) | rs4958881 | 150 430 429 | A | 4.6E-01 | 1.07 |  | 1.6E-01 | 0.88 |  | 1.6E-01 | 1.14 |  |  |  |  |  |  |  |  |  |
|  | rs3792783 | 150 435 925 | C | 7.5E-01 | 1.03 |  | 8.2E-01 | 0.98 |  | 3.1E-01 | 1.09 |  |  |  |  |  |  |  |  |  |
|  | rs2233287 | 150 420 290 | G | 5.7E-01 | 1.06 |  | 2.3E-01 | 0.89 |  | 4.0E-01 | 1.09 |  |  |  |  |  |  |  |  |  |
|  |  |  |  |  |  |  |  |  |  |  |  |  |  |  |  |  |  |  |  |  |
|  |  |  |  |  |  |  |  |  |  |  |  |  |  |  |  |  |  |  |  |  |
|  | **B. Case category vs Controls analyses. Evidence of association by case category** | | | | | | | | | | |  |  |  |  |  |  |  |  |  |
|  |  |  |  | **Dc** | |  | **Lc** | |  | **ACA+** | |  | **ACA-** | |  | **TOPO+** | |  | **TOPO-** | |
| **Chr.-gene)** | **SNP** | **BP** | **Min.** | **P** | **OR** |  | **P** | **OR** |  | **P** | **OR** |  | **P** | **OR** |  | **P** | **OR** |  | **P** | **OR** |
| 6p21(PSORS1C1) | rs3130573 | 31 214 247 | G | 2.6E-03 | 1.20 |  | 2.6E-03 | 1.14 |  | 2.3E-01 | 1,07 |  | 4.2E-06 | 1,25 |  | 1.2E-03 | 1.23 |  | 2.6E-03 | 1.15 |
| 6p21 (HLA-DQB1) | rs6457617 | 32 771 829 | C | 2.9E-12 | 0.65 |  | 3.2E-20 | 0.66 |  | 5.7E-27 | 0,54 |  | 5.3E-14 | 0,70 |  | 3.9E-18 | 0,57 |  | 8.7E-20 | 0,66 |
|  |  |  |  |  |  |  |  |  |  |  |  |  |  |  |  |  |  |  |  |  |
| 2 (RHOB) | rs342070 | 20 548 952 | C | 1.5E-01 | 1.11 |  | 1.1E-05 | 1.24 |  | 1.9E-03 | 1.21 |  | 1.1E-03 | 1.19 |  | 5.5E-02 | 1.15 |  | 5.1E-05 | 1.22 |
|  | rs13021401 | 20 552 000 | T | 1.5E-01 | 1.11 |  | 2.7E-05 | 1.23 |  | 5.7E-03 | 1.19 |  | 7.2E-04 | 1.20 |  | 3.7E-02 | 1.16 |  | 1.1E-04 | 1.21 |
| 5 (TNIP1) | rs4958881 | 150 430 429 | A | 1.5E-03 | 1.33 |  | 5.4E-03 | 1.20 |  | 5.6E-02 | 1.18 |  | 2.3E-05 | 1.35 |  | 1.4E-03 | 1.35 |  | 1.3E-03 | 1.24 |
|  | rs3792783 | 150 435 925 | C | 6.3E-04 | 1.33 |  | 3.9E-03 | 1.19 |  | 5.2E-02 | 1.16 |  | 8.1E-06 | 1.34 |  | 1.1E-04 | 1.39 |  | 1.5E-03 | 1.22 |
|  | rs2233287 | 150 420 290 | G | 7.6E-05 | 1.35 |  | 5.7E-05 | 1.25 |  | 3.0E-04 | 1.28 |  | 1.9E-06 | 1.33 |  | 1.8E-05 | 1.39 |  | 1.3E-05 | 1.28 |

£ Diffuse cutaneous (Dc) vs Limited cutaneous (Lc) forms; $anticentromere antibodies positive (ACA+) vs negative (ACA-); *anti-topoisomerase I positive
