# Supplementary material for: Genome-Wide Scan Identifies TNIP1, PSORS1C1, and RHOB as Novel Risk Loci for Systemic Sclerosis
Source: PLoS Genet. 2011 Jul 7;7(7):e1002091. doi: 10.1371/journal.pgen.1002091 (PMC3131285; doi:10.1371/journal.pgen.1002091)
Supplement: Supplementary file 4 [file pgen.1002091.s002.doc]

Supplementary Table 2 Results of conditional logistic regression analysis for 28 SNPs (P<10-3) in MHC region

						Controlling on							
						rs9275224		rs6457617		rs3130573		rs6457617 & rs3130573	
**Chr. 6p21 GENE**	**SNP**	**BP**	**A1**	**OR**	**P**	**P**	**OR**	**P**	**OR**	**P**	**OR**	**P**	**OR**
	rs12526481	30 854 612	A	1.29	4.2E-04	3.3E-03	1.24	3.1E-03	1.24	8.6E-04	1.27	5.8E-03	1.22
PSORS1C1/C6orf205	rs3130573	31 214 247	G	1.36	1.9E-05	2.1E-05	1.36	2.0E-05	1.36	-	-	-	-
TNXB	rs547154	32 018 917	T	0.66	4.4E-04	1.1E-02	0.74	1.2E-02	0.74	2.2E-03	0.69	4.1E-02	0.78
	rs541862	32 024 930	C	0.66	4.6E-04	1.2E-02	0.74	1.3E-02	0.74	2.2E-03	0.69	4.2E-02	0.78
	rs438999	32 036 285	G	0.66	3.9E-04	1.0E-02	0.73	1.1E-02	0.73	1.9E-03	0.69	3.7E-02	0.78
	rs429608	32 038 441	A	0.70	2.4E-04	7.7E-03	0.77	8.4E-03	0.77	2.1E-03	0.74	4.2E-02	0.81
NOTCH4	rs2071286	32 287 874	T	1.32	2.4E-04	6.3E-02	1.16	6.6E-02	1.16	2.5E-04	1.32	6.8E-02	1.16
BTNL2	rs28366174	32 374 468	C	0.58	7.8E-05	7.3E-03	0.68	6.5E-03	0.68	4.5E-04	0.62	2.6E-02	0.73
	rs28366191	32 472 168	G	0.60	3.4E-04	1.7E-02	0.71	1.6E-02	0.70	1.9E-03	0.64	5.9E-02	0.76
HLA-DQA1	rs13204672	32 690 774	G	0.66	1.2E-04	3.6E-03	0.72	3.2E-03	0.72	1.1E-04	0.66	2.6E-03	0.72
HLA-DQB1	rs9271348	32 691 720	G	0.75	4.6E-04	1.3E-01	0.87	1.2E-01	0.87	1.5E-03	0.77	2.1E-01	0.89
	rs9275224	32 767 856	A	0.69	9.2E-08	-	-	-	-	9.8E-08	0.68	-	-
	rs9275245	32 768 921	A	0.69	1.4E-07	-	-	-	-	1.5E-07	0.69	-	-
	rs5000634	32 771 542	G	1.28	4.3E-04	3.6E-01	0.91	3.5E-01	0.90	3.5E-04	1.29	4.0E-01	0.91
	rs6457617	32 771 829	C	0.69	1.1E-07	8.5E-01	0.92	-	-	1.2E-07	0.69	-	-
	rs9275371	32 776 274	C	1.31	4.5E-04	3.9E-01	1.08	3.4E-01	1.09	2.4E-04	1.33	2.5E-01	1.11
	rs9275374	32 776 504	T	1.32	3.9E-04	3.6E-01	1.09	3.5E-01	1.09	2.0E-04	1.34	2.5E-01	1.11
	rs9275388	32 777 062	C	1.31	4.7E-04	3.9E-01	1.08	3.5E-01	1.09	2.4E-04	1.33	2.5E-01	1.11
	rs9275406	32 777 933	T	1.31	5.1E-04	4.0E-01	1.08	3.6E-01	1.08	2.6E-04	1.33	2.6E-01	1.11
	rs2858308	32 777 978	T	0.61	4.8E-05	1.8E-02	0.73	1.6E-02	0.73	1.1E-04	0.62	2.8E-02	0.75
	rs9275407	32 778 015	T	1.32	4.4E-04	3.8E-01	1.08	3.4E-01	1.09	2.3E-04	1.34	2.4E-01	1.11
	rs9275418	32 778 222	G	1.32	3.5E-04	3.4E-01	1.09	3.3E-01	1.09	1.8E-04	1.34	2.3E-01	1.11
	rs9275425	32 778 852	A	1.32	3.6E-04	3.5E-01	1.09	3.2E-01	1.09	1.9E-04	1.34	2.3E-01	1.11
	rs2856705	32 778 934	T	0.61	4.8E-05	1.8E-02	0.73	1.6E-02	0.73	1.1E-04	0.62	2.8E-02	0.75
	rs13192471	32 779 081	C	1.47	1.8E-05	2.1E-02	1.25	2.2E-02	1.25	2.0E-05	1.47	2.3E-02	1.25
	rs9275439	32 779 499	C	1.32	4.2E-04	3.8E-01	1.08	3.4E-01	1.09	2.0E-04	1.34	2.4E-01	1.11
	rs10807113	32 830 164	C	0.78	4.4E-04	3.6E-01	0.93	3.5E-01	0.93	6.4E-04	0.79	4.2E-01	0.94
	rs7756516	32 831 895	C	0.78	4.1E-04	3.5E-01	0.93	3.4E-01	0.93	6.0E-04	0.79	4.1E-01	0.94
